# Appendicular perforation at the base of the caecum, a rare operative challenge in acute appendicitis, a literature review

**DOI:** 10.1186/1749-7922-6-36

**Published:** 2011-11-04

**Authors:** Chee Siong Wong, Syed Altaf Naqvi

**Affiliations:** 1 Department of Surgery, Mid Western Regional Hospital, Dooradoyle, Co. Limerick, Ireland

## Abstract

**Background:**

Acute appendicitis is the most common acute surgical condition of the abdomen. Diagnosis is made based on full clinical history and examination as well as supported by a routine blood investigation and urine test. Prompt diagnosis and surgical referral may reduce the risk of perforation and prevent complications. The mortality rate of non-perforated appendicitis is less than 1 percent. Perforated appendicitis is associated with a higher mortality rate - as high as five percent and may be particularly more in extreme of age group attributed to delay in clinical presentation or diagnosis in the younger group and multiple co-morbidities in the elderly group. The aetiology is unknown. It may be linked with lack of fibre, familial tendency, or viral infection. It may be precipitated by faecaliths. The commonest site of the appendix is retrocaecal.

**Case Report:**

We report a case of a 46 year old male who was admitted under the surgical service in Mid-Western Regional Hospital, Limerick with suspected appendicitis which turned out to be a perforated caecum, a rare complication of an acute appendicitis. We performed a literature review comparing two main approaches - right hemicolectomy and primary closure with omental patch - discuss and highlight their differences as well as a guide to its management.

**Conclusion:**

There are limited studies to compare these two surgical options in the literature. A larger prospective study is needed to compare both approaches and long term outcome.

## Background

Acute appendicitis remains the most common reason for intervention in acute abdominal pain. Diagnosis is made based on full clinical history and examination as well as supported by a routine blood investigation and urine test. It is a common condition can be difficult in making a diagnosis when the clinical picture is borderline suggestive of acute appendicitis. Especially in children, acute Meckel's diverticulitis must be kept in mind, as the clinical picture is indistinguishable from acute appendicitis. Perforation of a large bowel is associated with severe acute appendicitis but further surgical management of this condition uncommonly described in the literature. We highlighted this question and performed a literature review to compare two possible surgical approaches faced by surgeons.

## Case Report

A 46 year old man presented with a day history of sudden onset of right iliac fossa pain associated with nausea, fever, and anorexia. No urinary and bowel symptoms. There was no significant past surgical or medical history. No history of recent travel and family history of colitis or inflammatory bowel disease. On physical examination, his temperature was 39.4 degree Celsius, pulse rate 91 beats per minute, blood pressure 159/80 mmHg, respiratory rate 20. His abdomen was not distended but tender in the right iliac fossa with some voluntary guarding. No rebound tenderness was elicited on examination. Rovsing's sign was positive.

Full blood count shows elevated WBC 19.91 × 10^9^/L, Hb 13.7 g/dl, Platelet 242 10^9^/L. Na 137 mmol/L, K 3.8 mmol/L, urea 4.8 mmol/L, creatinine 92 mmol/L, amylase 24 IU/L. Urine Microscopy - negative for urinary tract infection, leucocytes < 10/ul and red cell < 10/ul.

**Figure 1:**
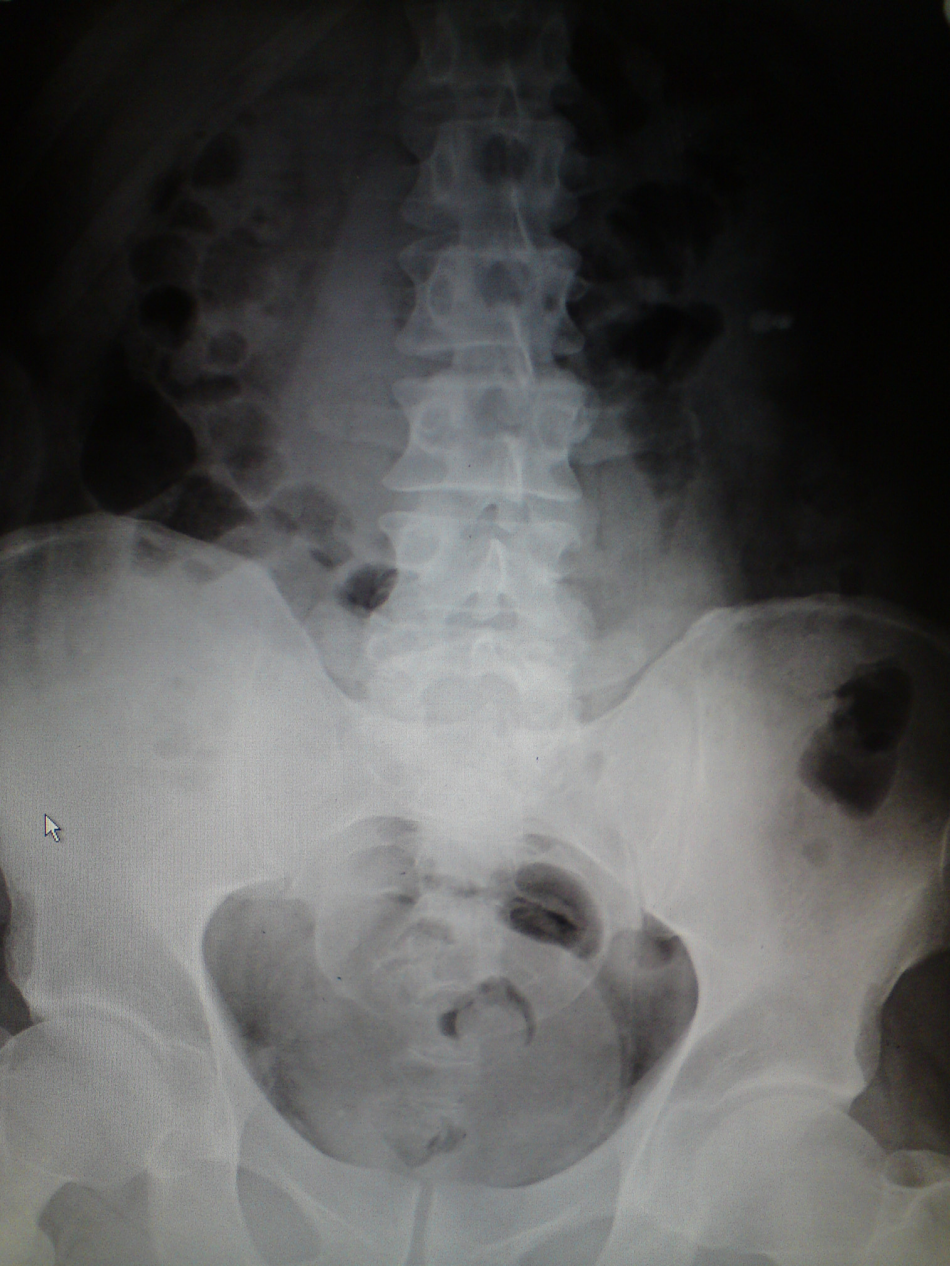
**Normal plain film of the abdomen**.

**Figure 2:**
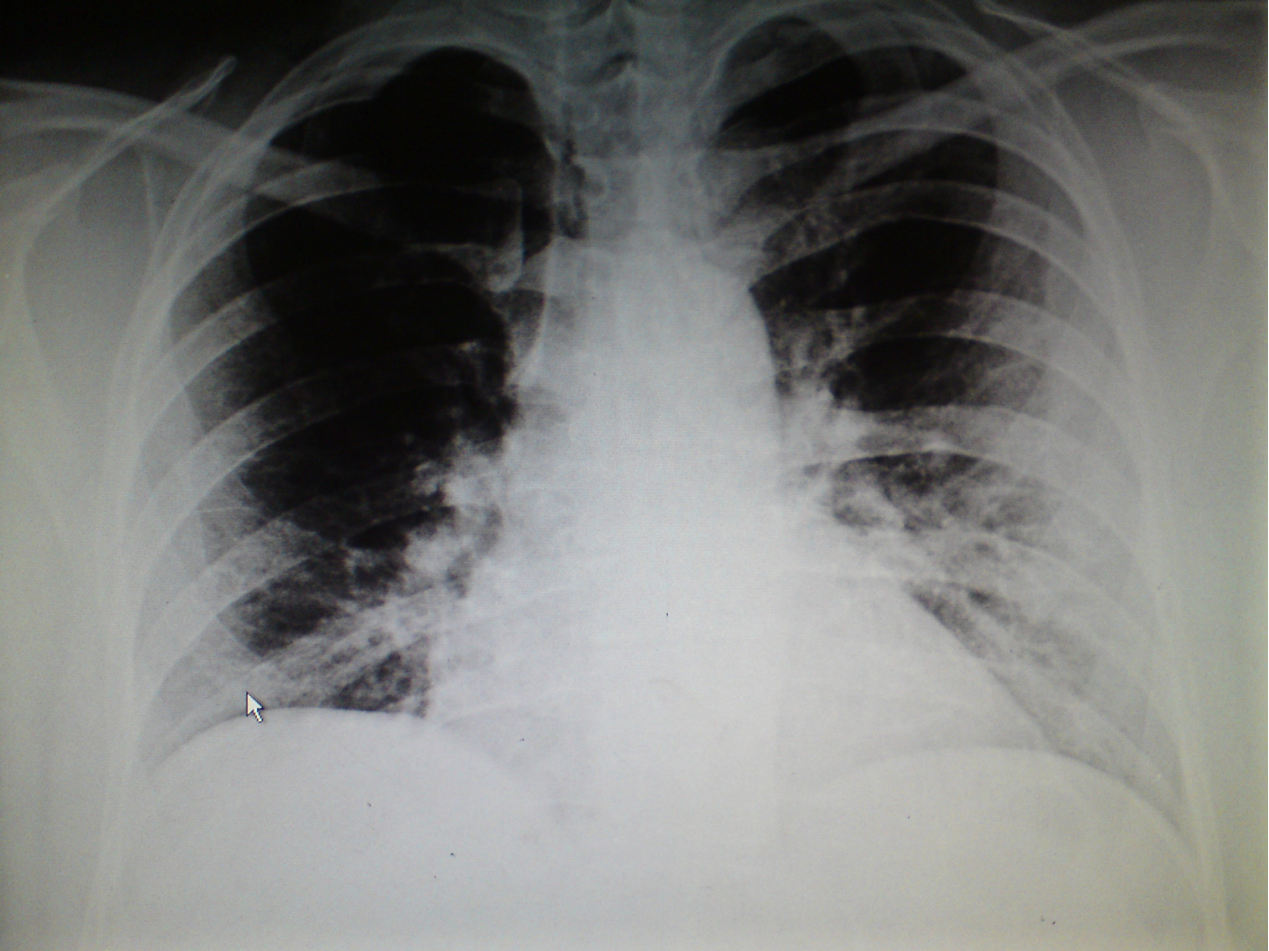
**Normal erect chest x-ray**. No air under the diaphragm.

Plain film of Abdomen and Chest X-Ray were not remarkable (Figure 1 and 2). Diagnosis of acute appendicitis was made clinically and the patient was consented for an open appendicectomy under general anaesthesia.

**Figure 3:**
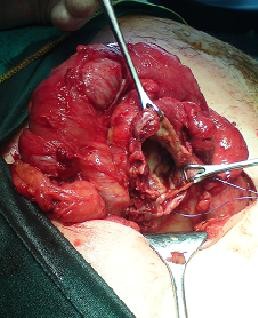
**A large perforation of the appendix at the base of the caecum**.

**Figure 4:**
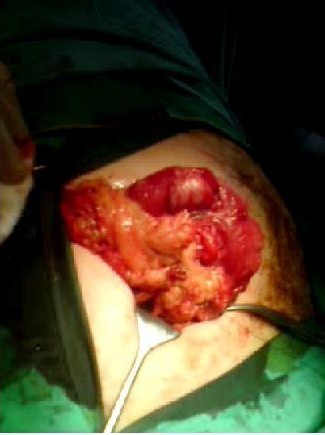
**The perforation was oversewn and omentum was used to cover the defect on the caecum**.

Operation: Intravenous antibiotics were commenced pre-operatively. An extended McBurney's or grid iron incision was made. Dissection of the appendix was carried out with some difficulties and approximately 50 mls of pus found in the peritoneal cavity around the appendix. There was a large 3 × 3 cm caecum perforation seen at the base of the appendix (Figure 3). Macroscopically, appendix was perforated and gangrenous. Perforation at the base of caecum was repaired with an absorbable suture and the omental patch was used to cover the caecum (Figure 4). A thorough washout with warm saline and bethidine solution was carried out to prevent gross peritoneal contamination. A corrugated drain was inserted. The abdominal incision was closed by a mass closure technique using loop PDS 2/0 and absorbable sutures to subcutaneous tissue and staples to skin.

Post operative progress. Inflammatory markers were responding with intravenous antibiotic. No further spiking temperature. The drain was removed postoperative day 5 and patient was discharged the next day. The histolopathology of the appendix showed acutely inflamed appendix with periappendiceal abscess formation. The epithelium shows reactive/reparative changes. No malignancy is seen.

## Discussion

Appendicitis perforations, commonly occur at the tip of the appendix, are associated with the presence of a faecolith on CT scan and not the anatomical location of the appendix (retrocaecal appendix) as previously thought [1]. Perforation of caecum is an uncommon differential diagnosis for an acute appendicitis. Other possible causes of caecum perforation include perforated right diverticulitis [2,3], caecal tumor, and rarely associated with foreign body [4,5], in burn patient [6], tuberculosis infection [7] and following caesarean section [8,9] or iatrogenic endoscopic procedure had been reported. Surgery for colonic perforation is associated with high morbidity and mortality rates.

While omental patch repair is a common surgical approach to management of stomach and duodenum perforation, there are only few reports in the literature that compare two very different surgical approaches - omental patch with primary repair vs right hemicolectomy. In the presence of an uncomplicated perforation, absence of severe infection, and well controlled localized haemostasis - a less invasive surgical approach with post operative intravenous antibiotics would be the management of choice.

Right hemicolectomy carries a higher morbidity and mortality but it is generally recommended only in selected cases - severe inflammation, torsion, haemorrhage, and inflammatory mass or caecal neoplasm found intraoperatively [10]. The presence of severe appendicitis; or caecum appears necrotic in some cases warrants right hemicolectomy to be performed.

A caecum perforation is a very rare identity and so far only nine case reports have been published (Table 1). The most frequent operation for perforated caecum is right hemicolectomy although some surgeons might advocate oversewn the perforation is equally adequate in repairing the defect. The advantages of the latter are associated with shorter length of hospital stay, less blood loss, easier haemostasis control, and lower risk of anastomosis breakdown. However, there is no clinical data yet to support this hypothesis.

**Table 1:** Various similar case reports and their surgical approaches

Author (Year) [Ref]	Case Reports
Jain et al (2010) [7]	Primary tubercular caecal perforation and a right hemicolectomy with ileostomy was performed

Cole et al (2009) [11]	A perforated caecal diverticulum and a right hemicolectomy was carried out

Papapolychroniadis et al (2004) [2]	Two cases of perforated caecum diverticulum and right hemicolectomy was carried out on both cases

Mauvais et al (1999) [3]	Perforated caecum due to diverticulitis on post operative findings. However author did not discuss further on surgical approach

Vitali et al (1998) [12]	Caecal perforated diverticulitis but did not mention of its surgical approach

Mosca et al (1997) [13]	A case of perforated caecum diverticulitis and right hemicolectomy was carried out

Ghoneim et al (1995) [6]	Caecal perforation in burn patient was treated using a right hemicolectomy

Dorfman et al (1990) [14]	Reported five cases of perforated caecal diverticulitis. Two cases were treated with a right hemicolectomy

Wesch et al (1980) [8]	Two cases of perforation of the cecum following caesarean section. The perforation is oversewn

Although right hemicolectomy may be the conventional approach in some cases of caecal perforation, however, in a highly contaminated case as such in this scenario would have a significantly higher postoperative complication likely secondary to infection or systemic septicaemia. Therefore, the decision for a primary repair of the perforation was carried out.

## Conclusion

A primary hemicolectomy in perforated lesion of the caecum is recommended but there have been no recent studies comparing this approach with primary caecum repair with omental patch. A larger prospective study is needed to compare both approaches and long term outcome.

## Competing interests

The authors declare that they have no competing interests.

## Authors' contributions

MW drafted the manuscript, searched the literature and the findings, manuscript writing & editing and submission of the manuscript. SAN critically reviewed the manuscript. Both authors read and approved the final manuscript submission.
